# Effect of Wave Process of Plastic Deformation at Forging on the Fatigue Fracture Mechanism of Titanium Compressor Disks of Gas Turbine Engine

**DOI:** 10.3390/ma14081851

**Published:** 2021-04-08

**Authors:** Andrey A. Shanyavskiy, Alexey P. Soldatenkov, Alexandr D. Nikitin

**Affiliations:** 1 Aviation Register for Russian Federation, Airport Sheremetievo-1, PO Box 54, 141426 Moscow Region, Chimkinskiy State, Russia; 106otdel@gmail.com; 2 Institute of Computer Aided Design, 2nd Brestskaya street 19/18, 123056 Moscow, Russia; nikitin_alex@bk.ru

**Keywords:** forged titanium alloy, low-cycle fatigue, cycle wave form, durability, crack meso-tunneling, fractography, crack growth duration

## Abstract

The low-cycle fatigue behavior of the VT3-1 titanium alloy (Ti–6Al–3Mo–2Cr alloy) under loading with a triangular and trapezoidal shape of cycle waveform was studied on round specimens prepared from forged compressor disks of a gas turbine engine. The filament type structure after forging has alternating filaments with the ductile and quasi-brittle state of the metal as a result of the wave process of plastic deformation during the metal forging process. The crack propagation, regardless of the cyclic waveform shape, occurs by the crack meso-tunneling mechanism: initially, the cracks propagate along the filaments by a quasi-brittle mechanism with the formation of a facetted pattern relief on the fracture surface reflecting the two-phase structure of the titanium alloy, and then, the bridge between the meso-tunnels is fractured with the formation of fatigue striations. The part of the crack growth duration *N*_p_/*N*_f_ in the durability *N*_f_ is determined on the basis of measuring the fatigue striation spacing, and it depends on the crack path with respect to the material filaments. The growth of a fatigue crack in the case of in-service failure of a compressor disk of a gas turbine engine is considered, taking into account the crack meso-tunneling effect, and the fatigue crack growth duration in the disk is determined on the basis of quantitative fractography.

## 1. Introduction

Compressor disks of gas turbine engines (GTE) are manufactured from various titanium alloys and operate under high-stress conditions. Therefore, the main criterion for determining their service life is low-cycle fatigue (LCF) [1,2]. A high-stress level of the disks is considered in repetitive cycles of variable loads, which represent a load block for a flight or a cycle of starting and stopping the engine. It should be emphasized that the LCF regime is considered in terms of the number of loading cycles within 4 × 10^4^ engine start and stop cycles in service (FLC—flight loading cycle). In this case, the stress level in zones with a high-stress concentration does not exceed the value of σ_max_ = 0.8 ∙ σ_0.2_, where σ_0.2_ is the yield stress of the material. Therefore, according to fatigue S-N curves, the service life of more than 4 × 10^4^ FLC can be realized. This requirement reflects the fact that the transition to the LCF regime according to the criteria of the physics of metals means that the yield stress of the metal is reached, which is associated with a change in the shape of the object, and such a case is not permissible for the compressor disk in the structure.

Plastic strains appear to be localized, but not leading to a change in the shape of the structure on the macro-scale level. Macroplastic strains, characterizing the LCF regime for the specimens, are not observed during the LCF regime of disks.

The problem of the fatigue crack development in compressor disks of gas turbine engines arose in the mid-1980s [1,3,4,5,6]. It is generally associated with the structural features of two-phase (α + β) titanium alloys and the pronounced occurrence of their sensitivity to dwell-time under load [6,7,8]. Cracks can appear both in the rim [4] and in the hub part of the disks [3,5,6]. Therefore, the fracture of disks is determined not by the presence of material defects but by the sensitivity of the material to operating conditions (long dwell-time under load) or the arising anomalous high-stress state, in most cases leading to the initiation and propagation of cracks in the LCF regime.

Analysis of crack growth regularities in fractured disks, mainly made of VT3-1 titanium alloy (Ti–6Al–3Mo–2Cr alloy), showed that the main feature of the fracture surface formation is expressed in a combination of two crack growth mechanisms [5,6]. Firstly, a quasi-brittle fracture occurs with the formation of a facetted pattern that reflects the geometry of the two-phase structure of the metal (globular or lamellar), and secondly, fatigue striations are formed, reflecting the ductile development of fracture typical of a material [5,6,9,10,11]. It is obvious that the combination of the two fracture mechanisms reflects a radical difference in the reaction of the material structure to the realized loading conditions of the disks during the full cycle of operation from start to stop of the engine. During the specified period of engine operation, the disk is affected by a sequence of loads varying in level and the number of unit cycles associated with the sequence of flight stages: taxiing the aircraft along the runway, takeoff, cruising, landing, and taxiing the aircraft. It is worth emphasizing that during the full flight loading cycle, the disks experience long-time loading with a constant level of stress, for example, in the cruising mode, which leads to an intensification of the fracture process, which was expressed in the formation of a facetted pattern.

Since the engines operate at modes with various numbers of revolutions due to different stages of a flight, not one but several cycles of material damage are considered. Hence, the actual number of cycles damaging the disks is increased up to five unit cycles for one FLC [4,12]. Therefore, when estimating the crack growth duration in terms of the number of engine start and stop cycles, it is necessary to take into account the actual number of damaging cycles based on the schematized block of cyclic loads.

Tests of one of the GTE compressor disks in air and at room temperature, as well as of specimens prepared from the compressor disk, using the triangular and trapezoidal shape of the loading cycle showed [4] that with an increase in the duration of the loading cycle and dwell-time up to 1 min, the durability and the crack growth duration decrease simultaneously. The decrease in durability is accompanied by a change in the mechanism of material fracture from ductile to quasi-brittle. In the first case, fatigue striations are predominantly formed, and in the second case, a facetted pattern of the fracture surface, reflecting the two-phase (α + β) lamellar or globular structure of the material, is formed [5].

Thus, in the existing technological cycle of disk manufacturing, when the mechanical characteristics of the material specified in the drawing and the chemical composition of the material meet the requirements imposed on them, the material may turn out to be both less and more sensitive to the conditions of its loading in operation.

To eliminate the inhomogeneity in the material structure formed during forging, the technology of the multiple and multidirectional disk forging process was proposed [13]. However, as shown by fractographic studies, in this case, even with a durability of 800 cycles for smooth specimens, cracks originate under the surface, which excludes their control in service [14].

Reducing the stress state of the disk or limiting their resource does not exclude the occurrence of new fractures due to the natural scatter of material properties in the rim or the hub part of the disks, as well as variations in the stress state of the material as a result of natural clearances in the area of the blade positions. Unfortunately, this leads to the appearance of fatigue cracks in the disks of different compressor stages at different operating times.

At the same time, the experience of studying similar compressor disks shows that the process of fatigue crack propagation in them is characterized by general regularities, which are expressed in the following: there is a pronounced process of alternating fracture surface areas with a facetted pattern, which reflects the structure of a two-phase titanium alloy, as a rule, lamellar or mixed lamellar-globular type; these areas alternate with areas of fracture surface with fatigue striations, the spacing of which can sharply change over a short fracture surface length; the areas are oriented in a certain direction, which is set by the orientation of filaments in the texture of the deformed material.

In order to introduce more advanced technological solutions into the disk manufacturing process, it became necessary to study in detail the mechanism of fracture development using the specimens from a titanium compressor disk. The results of a comprehensive study of specimens that were prepared from titanium compressor disks and subsequently tested in the LCF regime as well as a discussion of the fracture mechanism and an estimation of the crack growth duration in the titanium compressor disk of the GTE of the An-124 aircraft are presented below in a generalized form.

## 2. Materials and Methods

At the first stage of the research, the specimens that were prepared from the first-stage disks of the engine low-pressure compressor were tested. The disk operated for about 8000 flight cycles (FC) without violating the operating conditions. The specimen dimensions were calculated in accordance with the ASTM E606 [15]. The specimens have a uniform-gage test section with a diameter of 6 mm and gage length of 20 mm. In the grips area, the specimens had a larger diameter (12 mm) and threads to prevent them from slipping out of the grips.

Before preparing the specimens, all disks were identified as corresponding in terms of mechanical characteristics and chemical composition to the two-phase VT3-1 titanium alloy in accordance with the requirements of the drawing (Table 1). The chemical element content was evaluated by the method of an atomic emission spectroscopy.

The fatigue tests were performed on specimens according to the loading scheme with constant maximum strain at three levels 0.0065, 0.0073, and 0.008 with two waveforms of the loading cycle: (1) triangular, with a frequency of 0.5 Hz; (2) trapezoidal, with both loading and unloading times of 5 s and a dwell-time of 20 s at a maximum strain level. A total amount of 72 specimens were tested at the stress ratio *R* = 0.

After testing, all fractured specimens were subjected to metallographic, spectroscopic, and fractographic analyses.

**Figure 1:**
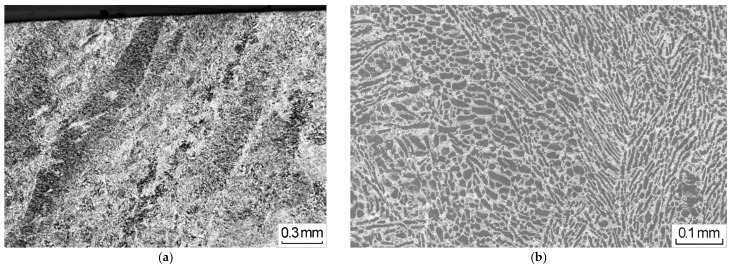
The microstructure of the two-phase (α + β) titanium alloy with a mixed type of globular and lamellar structure at (**a**) 0.3 mm magnification and (**b**) 0.1 mm magnification.

The metallographic analysis confirmed the correspondence of the specimen material to a two-phase (α + β) titanium alloy with a mixed type of globular and lamellar structure (Figure 1).

Energy dispersive X-ray analysis was carried out to identify the chemical composition for individual phases. Fractographic analysis of the fractured specimens was performed using the scanning electron microscope EVO-40 (Carl Zeiss GmbH, Oberkochen, Germany).

At the second stage of the study, a fractographic analysis of the regularities of fatigue crack propagation in the rim of the disk of the intermediate-pressure compressor (IPC) of the GTE was carried out.

**Figure 2:**
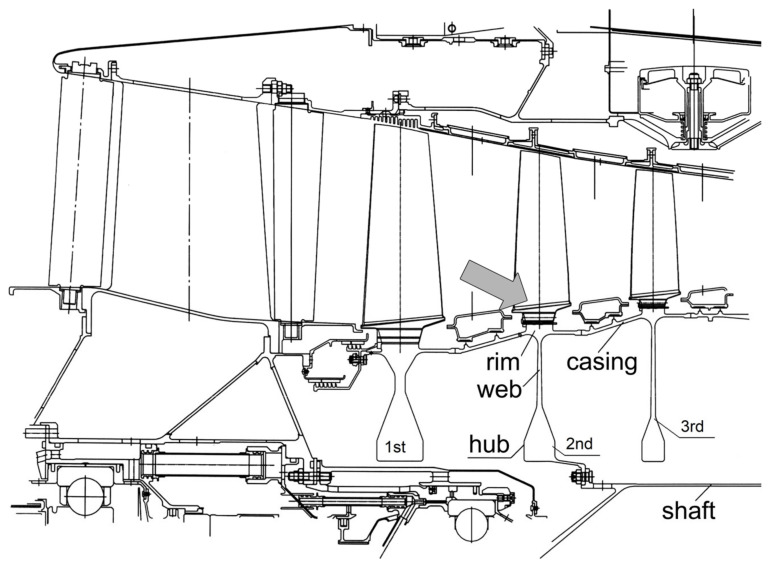
Fragment of the scheme of the GTE intermediate-pressure compressor. The arrow indicates the location of the initial fracture of the second-stage disk.

During the operation of the An-124 aircraft with the number RA-82043, a surge of the GTE of the power plant No. 4 occurred in the climb regime, with a fire alarm and automatic activation of the first stage of the fire extinguishing system. Subsequently, the engine No. 4 was automatically shut down. The aircraft landed safely at the departure airport. Inspection on the ground of the failed engine established its non-localized fracture due to the release of fragments of the titanium second-stage disk of the IPC (Figure 2).

The engine operated for 14,986 h (3299 FC) since the beginning of the operation, including 11,191 h (2486 FC) after the last overhaul.

For all specimens and the disk fracture surface, the duration of fatigue crack growth was estimated based on measuring the spacing of fatigue striations in the direction of crack propagation, regardless of the striation orientation [1,14].

## 3. Results and Discussion of Fatigue Tests

**Figure 3:**
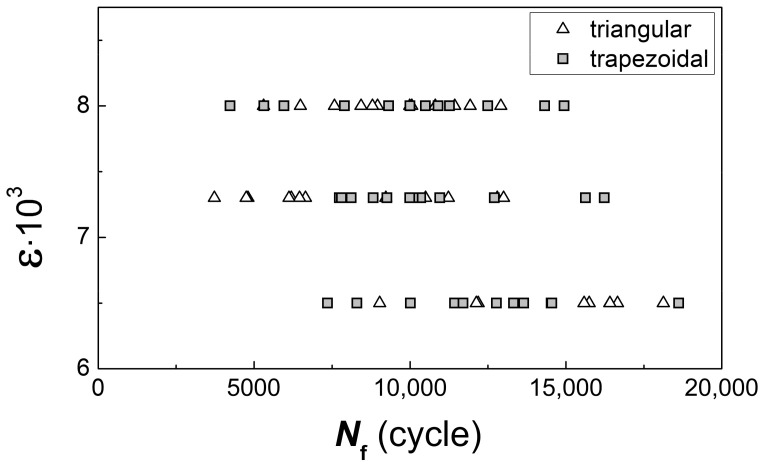
Fatigue ε-*N* curve plotted for tested specimens with different material behavior. The specimens with a life *N*_f_ of 7000 cycles and less showed sensitivity to loading conditions.

The test results are shown in Figure 3. In the specimens tested with different shapes of the cycle waveform, different fracture surface patterns were formed, in which the behavior of the material corresponded to the first, second, or third type according to the classification considered below.

The cracks in all the specimens initiated from the scores (<5 μm) made on the surface during the manufacturing procedure of the specimens. Subsequent crack propagation occurred along, across, or at an angle of less than 90° to the material filaments.

A fundamental difference in the durability of the specimens due to the presence of scores with different shapes and depths was not obtained. For example, for a specimen with high durability, the depth of the score differs a little from a specimen that has fractured with significantly lower durability with the same type of fracture surface pattern.

The feature in the behavior of the material, which is sensitive to the loading conditions, was revealed in a durability decrease; in some cases, the durability of such specimens was almost three times less than for the majority of specimens tested with the same strain range (see Figure 3). The decrease in durability was related to the dominance of the quasi-brittle fracture surface pattern with the formation of a predominantly facetted pattern of a fracture surface and few local zones with fatigue striations.

**Figure 4:**
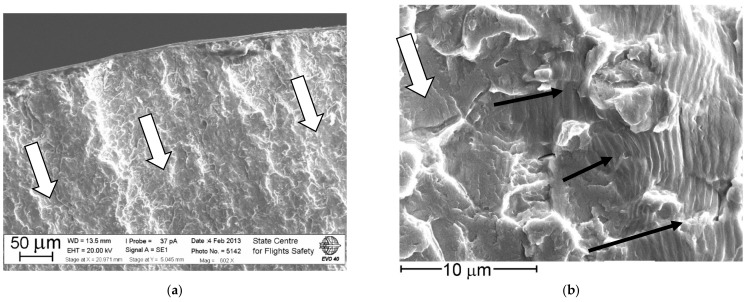
Meso-tunnels (indicated by hollow arrows) near the fracture origin (**a**) and at a several distance (**b**) in the crack growth direction for specimen tested at the trapezoidal shape of cycle waveform with ε_max_ = 0.0073 and *N*_f_ = 10,902 cycles. Dark arrows indicate the direction of crack growth during the formation of fatigue striations.

This concerns primarily specimens tested with the triangular cycle waveform at a maximum strain level of 0.0073. In the fracture surfaces, the filament type structure of the material was revealed, inherited from the forging of disks. A similar situation was found in some specimens tested with the trapezoidal shape of the cycle waveform at the same maximum strain level of 0.0073 (Figure 4). The crack propagated in the specimen along the filaments with pronounced tunneling and the formation of a predominantly quasi-brittle fracture surface relief. The connection of meso-tunnels along the bridge between them led to the formation of local areas with groups of fatigue striations oriented almost perpendicular to the main direction of crack growth (Figure 4b). It follows that in those zones of crack propagation where the quasi-brittle fracture occurred, the material had a reduced resistance to crack growth. During loading, the crack initially propagated along the filaments on the uploading part of a cycle, while during the formation of fatigue striations, the crack grew on the unloading part of a cycle [16].

Depending on the loading conditions and the stress state of the alloy, three types of fatigue crack propagation are possible under cyclic loading conditions.

**Figure 5:**
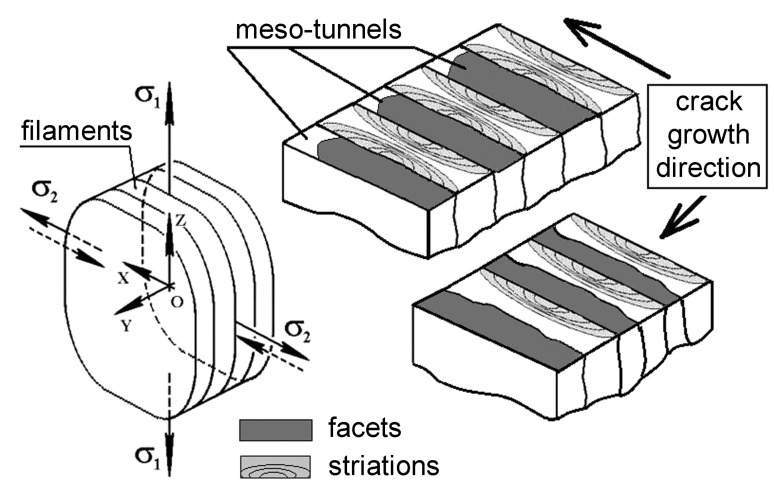
Scheme of the biaxial stress state of the material after forging and the difference in fracture surface formation along brittle and across ductile filaments at different directions of crack growth.

First type. The crack initially propagates along the brittle filaments with the formation of a facetted pattern of fracture surface and meso-tunnels, as shown in Figure 5. Then, the crack develops across the ductile filaments by the bridge fracture with the formation of fatigue striations.

Second type. The crack propagates perpendicular to the filaments with an alternating waved pattern of a fracture surface and zones with fatigue striations (see Figure 5).

Third type. The crack grows at an angle to the elongated zones with filaments, which leads to an alternation of the fracture process according to the first and second variants.

Nevertheless, fatigue striations were observed on all fracture surfaces with a different fraction of the whole fracture surface area.

The performed local X-ray analysis showed that in all zones of fracture surface, the similar homogeneity in the distribution of alloying elements is systematically repeated. On average, the distribution of chemical elements in both phases of material meets the requirements of the technical specifications for the VT3-1 titanium alloy.

The distribution of chemical elements in phases within the slice is such that Mo is not detected within an accuracy of 0.5% in the α-phase, and its content in the β-phase is higher than the grade composition, which meets the requirements of the alloy grade composition, since Mo is a β-phase stabilizer. Cr is typically present in the β-phase and may not be observed in the α-phase.

**Figure 6:**
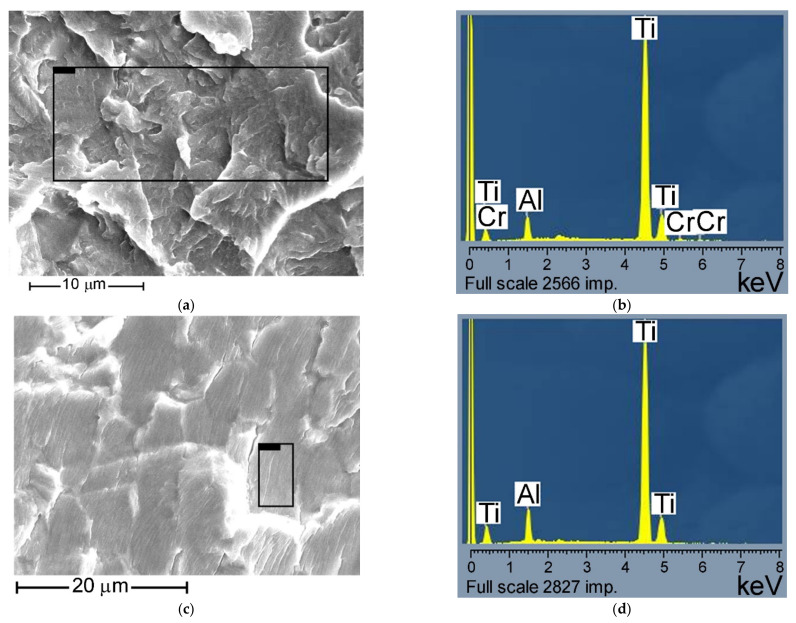
(**a**) Region of fracture surface with quasi-brittle pattern; (**b**) the spectrum of the main alloy elements distribution over the rectangular area of fracture surface on (a); (**c**) region of fracture surface with striations; (**d**) the spectrum of the main alloy elements distribution over the rectangular area of fracture surface on (c).

The indicated distribution of chemical elements does not affect the regularity of the formation of a particular fracture relief along the α-phase (Figure 6). No fundamental differences in the chemical composition of the alpha phase are observed during the formation of both facetted patterns and relief with striations. Therefore, there is no phase difference in chemical composition for different types of fracture surface relief. The difference in the crack growth mechanisms is determined only by the local plastic characteristics of the material state.

Fractographic studies made it possible to conclude that the material of the tested specimens had a high inhomogeneity in resistance to the action of loads with various cycle shapes. The obtained durability scatter reveals the influence of two factors on material behavior.

First, in all specimens on the surface, there are scores of different depths (<5 μm) created during the manufacturing process. This creates for all specimens statistically the same inhomogeneity in the stress state of the surface in the crack initiation zones and affects the scattering of durability by the criterion of crack initiation, underestimating its value in relation to the behavior of smooth specimens.

Second, in a small group of specimens, a sharp decrease in durability is observed since the material sensitivity to loading conditions has a dominant influence on durability.

The data on fatigue tests of fractographically investigated specimens illustrates the conclusions (see Figure 3).

A group of specimens with the durability of 7000 cycles and less should be characterized by a single fatigue curve, the parameters of which indicate significantly lower durability of the material at the considered strain levels. A group of specimens with a life of more than 10,000 cycles should be characterized by a different fatigue curve. In fact, a bimodal distribution of fatigue life is considered when a small part of specimens in which the material is sensitive to loading conditions determines the minimum allowable resource for the entire set of tested specimens. Therefore, for in-service disks, there is a risk of failure with a low operating time of single instances, the material of which is in an unsatisfactory state from the point of view of the criterion under consideration; i.e., the sensitivity to loading conditions is revealed and consisted in predominant fracture with the formation of a facetted pattern even for a triangular shape of the cyclic load waveform.

The results obtained indicate the statistical inhomogeneity in the material behavior within one disk from one zone to another and also from one disk to another, since the specimens studied were made from different disks as well as from randomly selected zones of one disk.

Thus, the material sensitivity to the in-service loading conditions is not a characteristic of manufacturing a particular disk. In different areas of the disk, the sensitivity is revealed differently for the same forging process. Therefore, in the existing technology of disk manufacturing, disk samples can be obtained with different zones according to the material sensitivity to loading conditions, when the durability of the disks can be more than three times reduced as compared to most other disks that do not have the specified sensitivity.

Based on the performed statistical analysis of the chemical element distribution over all investigated fracture surfaces, one can conclude that the realized mechanisms of the facetted pattern or the fatigue striation formation in the α-phase is not associated with the local redistribution of chemical elements, but it is determined by the plastic properties of the metal after its deformation (i.e., during manufacturing). In local regions where the plastic deformation process can occur in the material, fatigue striations are formed. In the zones where the plastic deformation has been exhausted at the stage of manufacturing the disk, the formation of the facetted pattern relief on the fracture surface is realized.

As a result of measuring the distance between the fatigue striations, an ambiguous relationship between the crack growth duration and the specimen durability was revealed. First of all, this is argued by the difference in the critical crack lengths at which the final fracture is realized.

**Figure 7:**
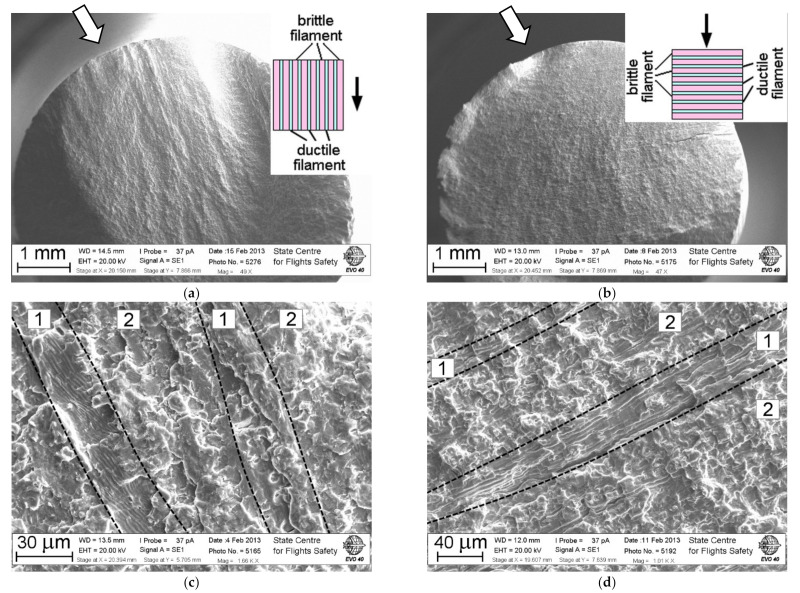
Fracture surfaces with the sketch of crack propagation (**a**,**c**) along and (**b**,**d**) across the filaments in a two-phase (α + β) structure of specimens tested with a triangular shape of the cycle waveform and the durability *N*_f_ of 16,566 cycles and 64,860 cycles, respectively. The dark arrow on the sketch indicates the direction of crack growth. The fatigue crack initiation zone is shown by the hollow arrow. Regions 1 and 2 correspond to fracture of ductile and brittle filaments, respectively.

In specimens where the stable crack growth with the formation of fatigue striations was realized almost for the total cross-section, the behavior of the material should be considered as the most favorable from the viewpoint of its structural (stress-strain) state. However, in several specimens, even with the predominant formation of fatigue striations, there was a sharp transition from the zone of stable crack growth to the fast fracture. As a rule, in the specimens, the cracks grew initially across the material filaments following propagation along the filaments. Such a sharp transition is associated with the fact that the crack began to develop not only within the plane of the main fracture but also along the filaments of the material, almost perpendicular to the plane of the fracture. This indicates that in some filaments, the material behaves as quasi-brittle, and the propagation of a crack through them is not accompanied by the formation of fatigue striations (Figure 7). In other filaments, the material is more ductile, and the propagated crack leads to the formation of fatigue striations.

In fact, in the process of forging, the disk material is formed as inhomogeneous with alternating filaments, within which the plastic deformation is significantly exhausted, and filaments can still realize significant plastic deformation in the process of fatigue crack growth. In the process of forging, individual grains of the material can be self-organized in volume with the formation of a filament type texture in such a way that it is in the grains that a facilitated sliding will be realized along the system of planes along which the subsequent crack development will predominantly occur. In this case, the material is already prepared for the implementation of the quasi-brittle fracture mechanism in the α-phase along the slip planes.

It is also necessary to take into account the natural variation in the crystallographic orientation of the α-phase within the filament group with relation to the realized loading [17]. In the case of significant exhaustion of plastic strain in the material along a particular group of filaments with an unfavorable orientation regarding the applied force, the faster initiation of a fatigue crack occurs. Then, transition to the fast fracture zone along the boundaries of the metal filament texture appears. This determines the scatter in the critical crack lengths.

**Figure 8:**
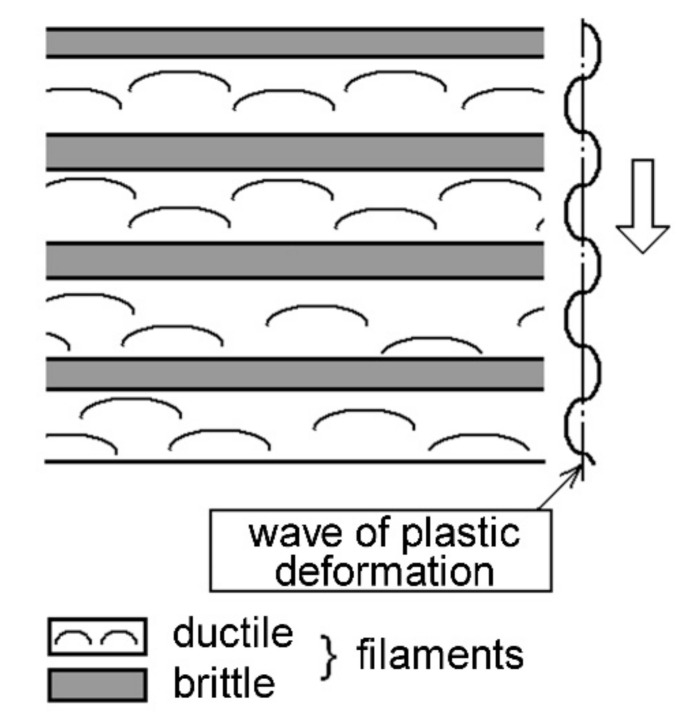
Scheme of the wave process of material deformation during forging with the formation of alternating filaments with a brittle and ductile material state.

Thus, during the disk forging process, an inhomogeneous wave process of plastic deformation occurs [18], which leads to the creation of a composite material with different levels of residual plastic strain along the formed layers (Figure 8). The deformation waves create periodically alternating filaments with a high and low degree of strain. The crack development along the filaments or a change in the orientation of the crack growth retains the difference in the fracture mechanisms for brittle and ductile filaments. However, due to the change in the crack orientation in the field of the biaxial stress state, the fracture of the brittle filaments occurs more ductile and without the formation of a facetted pattern [9,13,19], namely: for a positive λ-ratio, the fracture is more brittle than for a negative λ-ratio, where λ is the ratio of the first and the second principal stresses. Therefore, in the case of deformation of a two-phase titanium alloy during manufacturing in the material, the process of self-organized distribution of the residual stress level occurs due to the wave nature of the distribution of the intensity of the deformation effect on individual structural elements created during forging as a filament-type structure. Subsequent heat treatment does not lead to a uniformly prepared structure and the removal of stress state inhomogeneity in the material, which is due to a distinct difference in the plasticity of the formed individual filaments. In fact, the introduction of a dwell time under cyclic loading conditions for an inhomogeneous material creates conditions for its self-organization, which are expressed in the implementation of various fracture mechanisms.

Due to the existing difference in the material response to cyclic loading at the stage of crack nucleation and growth, with the same strain level and different orientation of initial crack propagation according to one of the above-mentioned types, it becomes necessary to estimate the crack growth duration as a component of the part in durability. For this purpose, the *N*_p_/*N*_f_ ratio between the crack growth duration *N*_p_ and the durability *N*_f_ was used.

In alloys on different bases, a stable and unambiguous relationship between the values of *N*_p_/*N*_f_ and *N*_f_ in the case of a statistically homogeneous state of the material exists [20,21,22]. In the case of specimens with a pronounced anisotropy in the material structure in the form of filaments, this relationship may be ambiguous due to a scatter of durability.

**Figure 9:**
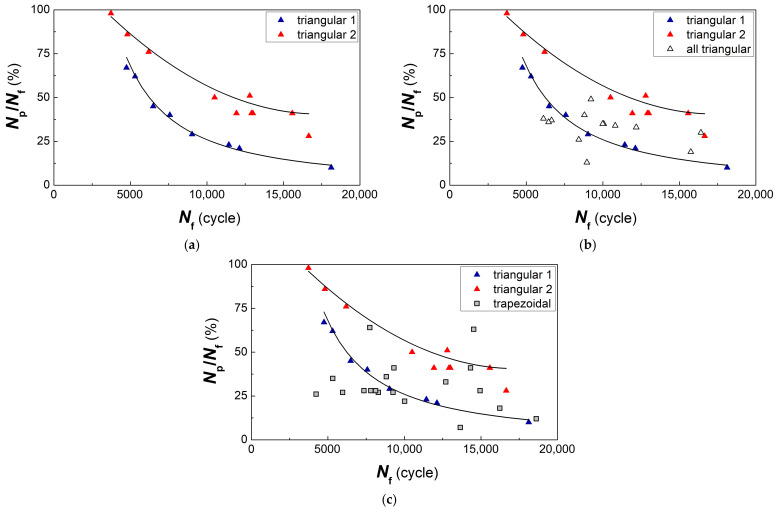
(**a**) Two groups of dependences 1 and 2 of the relative part of the crack growth duration *N*_p_/*N*_f_ on the durability *N*_f_ for specimens tested with the triangular shape of the cycle waveform, and the general dependence of changes in the relative fatigue crack growth duration on the durability for all specimens tested with (**b**) triangular and (**c**) trapezoidal shape of cycle waveform.

Therefore, initially, for each strain level and the triangular cycle waveform, the research results for specimens with minimum and maximum durability from the group located at the boundary of the scatter region are considered (Figure 9). It was established that the specimens tested at the same shape of cycle waveform belong to two different groups, for which the regularities of the change in the crack growth duration in relation to the realized specimen durability are different. One group of specimens in its behavior fits into the regularity of the crack growth duration, which was obtained earlier for the investigated specimens of the VT3-1 titanium alloy [1]. This analyzed dependence belongs to the largest values of the crack growth duration (Curve 2 in Figure 9). In this case, the crack propagated across the filaments, and ductile fracture with fatigue striations was mainly realized.

Another group of specimens indicates a smaller part of the crack growth duration in specimen durability (see Curve 1 in Figure 9). The surface of these specimens was possibly more hardened during their manufacturing than the surface of other specimens. Nevertheless, at the stage of crack propagation, faster fracture as compared to other specimens with approximately the same durability occurred due to the low resistance of the material to fatigue crack growth. It means that these specimens belonged to the first type of crack growth mechanism with the formation of meso-tunnels.

With a decrease in durability, starting from 7000 cycles, the data on relative crack growth duration approach each other. This indicates that at a high-stress level and fatigue life of 4000 cycles and less, the differences in the material structure of the specimens are not revealed significantly in the relative part of the crack growth duration.

Accounting for all the specimens tested with the triangular shape of the cycle waveform showed a significant scatter of the experimental data relative to the two dependences presented (see Figure 9b).

For a group of specimens tested with a trapezoidal waveform of the cycle, a similar analysis was performed. Some part of the specimens appears to have a similar difference in their behavior, and the relative part of the crack growth duration is grouped around the two dependencies on durability identified for the case of the triangular cycle waveform (see Figure 9c). This is quite natural, since the specimens were prepared under the same conditions, and their surface condition is statistically uniform.

It is necessary to point out a group of specimens with a life range of 4000–8000 cycles (on Figure 9c), which does not fit into the presented regularities. For this group of specimens, the relationship between the relative part of crack growth and specimen durability is not observed. It is the specimens that showed their sensitivity to the loading conditions, and at the stage of crack growth, a predominantly facetted pattern of quasi-brittle fracture with pronounced meso-tunneling of fatigue cracks was formed.

Two obtained dependencies (see Figure 9) confirm the fact that the tested specimens demonstrate a set of material states, one of which is bad, when already with a triangular waveform of the cycle, rapid nucleation and a short crack growth stage occur. Specimens showing reduced fatigue resistance during the crack growth stage should be classified as medium or also bad specimens. The arising wave process of plastic deformation [18], the scheme of which is shown in Figure 8, is responsible for such a material behavior. A good state will be observed when, in any direction of crack propagation, even in the material with weakly revealed filaments, the fracture mechanism associated with the formation of fatigue striations will dominate.

The established regularities of the metal texture formation during forging the disks allowed us to explain the observed regularities of the in-service compressor disks’ fracture process, which are demonstrated below on the instance of the GTE compressor disk fracture.

## 4. Fracture of the In-Service IPC Disk

### 4.1. Crack Growth Mechanisms

**Figure 10:**
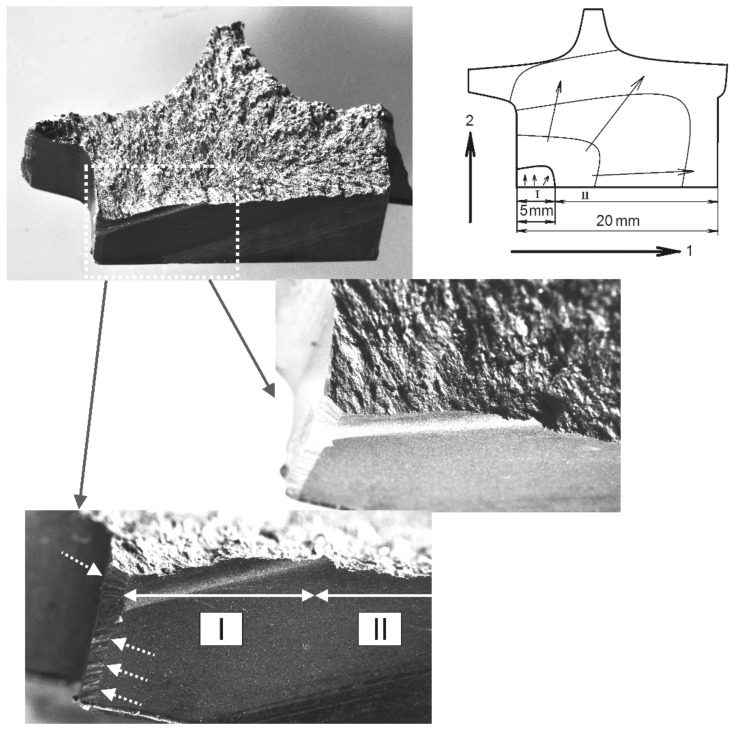
Initial fracture zone of the second-stage disk and its schematic representation indicating the orientation of the fracture surface formation from the region of the slot for the blade along the radius transition with the origin of the fatigue crack (indicated by a rectangle) in the disk. Dashed arrows indicate a region with high roughness along the boundary of the transition of the bottom of the slot for the blade to the rim surface. Roman numerals designate successively formed different zones of the fracture surface.

Based on the disk fracture surface analyses, it was established that the main fracture occurred in the radial direction from the rim part toward the disk hub (Figure 10). The initial zone of crack initiation and growth within about 5 mm from the lateral surface of the rim (zone I in Figure 10) is located along the slot for the blade. The fracture surface boundaries are oriented according to the scores from machining the surface along the radius transition of the bottom of the slot for the dovetail blade to the lateral surface of the inter-slot volume. Therefore, in the crack initiation zone along the slot for the blade along the radius transition, a combination of radial and tangential loads took place. Analysis of the crack initiation zone shape, its location, and changes in the path of fracture development at the subsequent stage shows that the crack initiation in the disk is generally due to the local stress concentration in the shape of the radius transition of the slot for the blade, and not to the total stress intensity of the disk.

**Figure 11:**
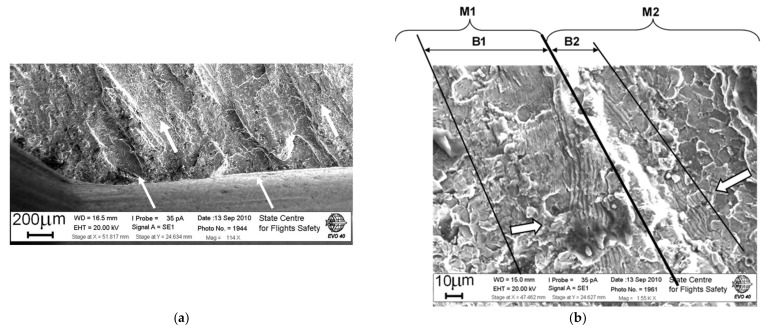
(**a**) Features on the zone I (see Figure 10) of fracture surface in the bridge between the initial meso-tunnels and (**b**) two meso-tunnels M1 and M2 with a facetted pattern. Blocks of fatigue striations formed simultaneously to meet each other are designated by B1 and B2, and the directions of fracture development are shown by hollow arrows.

It is established by the analysis of the fracture surface relief in the region I that the fracture has a multi-origin fatigue nature with the formation of a meso-tunnels’ cascade (Figure 11). In the fracture surface origins, a predominantly facetted pattern was observed. Between them, the blocks of fatigue striations were formed during the fracture of the bridges between the meso-tunnels. The uniformity of the fatigue striation formation has such a manner that within their block, the spacing remains almost the same. However, in some regions of the fracture surface (Figure 11b) within one meso-tunnel, there was a sharp change in the block of fatigue striation spacing of the same size to a block with a wider spacing. This reflects the specifics of the changing stress state of the material in the bridges between the meso-tunnels.

The observed nature of crack propagation with the formation of two types of fracture surface relief is typical for a material with a pronounced filament texture when the crack growth process is initially realized as a result of crack extension by the mechanism of a facet formation. Then, the bridges between the previously formed fracture surface regions are connected with the formation of fatigue striations (see, for example, Figure 4). In case of fracture from multiple origins, the duration of crack propagation in the direction 1 (see scheme in Figure 10) should be evaluated only starting from a distance of about 5 mm, when the crack emerged from the radius transition of the slot bottom under the blade (region II in Figure 10). This approach is based on the observation that on fracture surface region I in the direction 1, fatigue striations are formed toward each other (Figure 11b).

In the directions 1 and 2, the crack development took place with different regularity in the formation of fatigue striation spacing. The spacing of striations already in the zone I of crack initiation (see Figure 10) is 0.6 µm, which characterizes the process of initiation and development of disk fracture in the LCF regime. Along the initial fracture region with a width of about 5 mm in the direction 1, the fatigue striation spacing is almost constant. Further along the length of the crack in the direction 1, an increase in the striation spacing is observed. However, at a crack length of about 12 mm in the direction 1, the process of dimple formation increases, which indicates a transition to the unstable fracture zone of the disk material.

The crack has passed through a longer distance in direction 1 than in direction 2 during the same duration of disk fracture development. The size of the fracture zone in the direction 2 was about 10 mm, which is two times less than in the direction 1. Therefore, in the direction 1, the crack in the range of 12–20 mm grew at a higher rate than in the direction 2 for the same number of disk loading cycles.

The analysis of the regularities of the formation of cracks revealed during non-destructive testing in other slots for the blades showed the following. Fracture surfaces of the opened cracks in the rim part are formed similarly as in the above-described main fracture zone with the location of origins in the zones of the radius transition of the slot bottom for the blade to the surface of the inter-slot volume. In all fracture surfaces, the quasi-brittle fracture relief elements were observed, which are similar to the main fracture. However, the fracture origins are located only along the radius of transition from the surface of the slot for the blade to the inter-slot volume.

The observed relief elements indicate the development of secondary cracks in radius transitions along the inter-slot volume of the disk according to the LCF mechanism. These cracks appeared, most probably, after the LCF crack began to develop along the main fracture zone and had spread to a considerable depth. This is indicated by the fracture surface pattern along secondary cracks with a pronounced fast fracture process, the small length of the fatigue fracture zones, as well as the abrupt boundary of the transition to the fast fracture zone, formed in-service. In addition, fatigue striations in the zones were hardly pronounced.

According to the scheme of the main fracture surface formation (see Figure 10), the crack growth duration was estimated along the above-indicated directions 1 and 2. In direction 1, the estimation of the crack duration was important, because it was in this area that the crack extended to the greatest length over the surface (about 20 mm). However, as shown by the results of fractographic studies, it was in this area that the crack developed in such a manner that several meso-tunnels were formed simultaneously over a length of 5 mm, which were interconnected with the formation of fatigue striation blocks. This is indicated by the slightly different fatigue striations spacing, being in the range of 0.6–1.0 μm. The noted feature of relief formation is additionally illustrated by a fragment of a fracture surface with a pronounced process of crack development with almost the same fatigue striation spacing in the bridge between the meso-tunnels at the initial stage of the fracture zone formation (see Figure 11). From the facts considered above, it follows that the initial fracture zone with a length of 5 mm in the direction 1 should be excluded from the estimation of the crack growth duration, which underestimates slightly the total number of cycles during which the fatigue crack propagated. Such an underestimation goes into the safety margin and is acceptable for the practical implementation of non-destructive testing.

**Figure 12:**
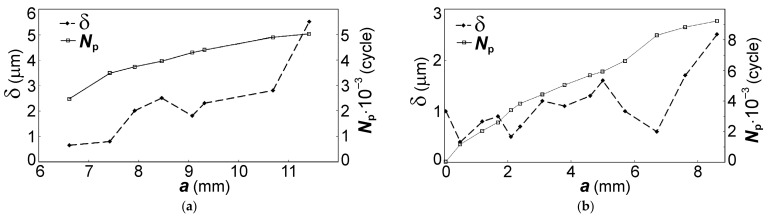
Dependences of fatigue striation spacing δ and the number of cycles for the crack propagation *N*_p_ on the crack length *a* in the rim of the disk for direction (**a**) 1 and (**b**) 2.

Therefore, in the direction 1, the regularity of fatigue striation formation was analyzed with the recalculation of the established number of unit cycles into the number of flight cycles on the length range of 5–20 mm. For example, the method of determining the crack growth duration by the fatigue striation spacing is shown in [14]. The fatigue striation spacing was measured, the number of loading cycles for the rim part of the disk during the fatigue crack propagation was calculated, and the results are shown in Figure 12. The increase in the fatigue striation spacing along the crack length was established to be insignificant. Stable formation of areas with fatigue striations occurred in the crack length range of 5–11 mm. Then, along the considered direction, an accelerated, repeated-static fracture of the disk was realized with the formation of areas with the facetted patterns and dimples. The crack growth duration resulted from measurements of the fatigue striation spacing at a length of 5–11.5 mm is about 5000 cycles and an additional 1900 cycles at a length of 11.5–20 mm according to the approximation of the spacing dependence on the length obtained at the interval of 5–11.5 mm.

As indicated above, the presented estimation is not complete, since it does not include the crack growth within the first 5 mm at the stage of origination zone formation. Therefore, the regularities of crack growth in the direction 2 (see Figure 10) were analyzed.

The results of the performed measurements showed that in this direction, from the corner of the fracture surface and before the transition to the fast crack growth zone with the formation of facetted patterns and dimples on the fracture surface, the crack development was obtained for a longer duration (Figure 12b). This indicates the fact that for the crack length range of 0–5 mm in the zone I, the formation of multiple fracture origins occurred and the initial stage of crack growth was quite long. Comparison of the crack growth duration values obtained for the two considered directions 1 (6900 cycles) and 2 (9600 cycles) showed the following. The duration of the fracture process for the region of 0–5 mm was at least 30% of the total crack growth duration from the initiation up to the critical length.

### 4.2. The Number of Aircraft Flights with a Developing Crack

**Figure 13:**
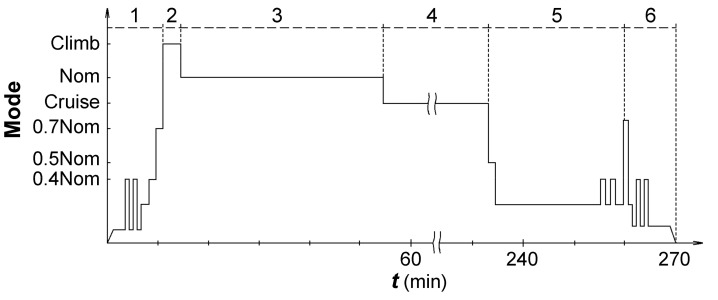
The schematized flight cycle of the GTE concerning with engine speed of revolutions relative to flight stages: 1—start-up, warm-up, taxiing (10 min); 2—take-off (3.5 min); 3—climb (40 min); 4—cruise (180 min); 5—descending (27 min); 6—reverse, engine cooling, landing, taxiing, engine shut-down (10 min). “Nom” is the nominal speed of revolutions.

The results obtained for evaluating the crack growth duration in terms of unit loading cycles allow one to proceed to the analysis of the crack growth duration in flight cycles. To make the conversion, let us consider the disk loading when the engine is running according to the stages of a typical flight of the An-124 aircraft (Figure 13). The disk loading should be considered at least in five stages of changing the engine operating modes, resulting in the propagation of a fatigue crack for a flight: starting and entering the operating mode, changing the mode of operation when maneuvering, as well as the stages of changing the mode of operation before landing. This is consistent with the previously performed analysis of schematized loading cycles for other types of engines with the introduction of recalculation of the crack growth duration in terms of unit cycles into flight cycles [1,4]. The above estimation of the crack growth duration in terms of unit cycles should be reduced by a factor of 5.

In addition, it is necessary to take into account the delay in the development of the crack estimated by both the fatigue striations and the crack growth rate along the disk surface. This discrepancy leads to the usage of a factor of 1.6 and reduces the calculation results based on measuring the striation spacing when recalculating into the number of flight cycles [1,4,12]. Therefore, the recalculation of the number of unit cycles *N*_p_ into flight cycles *n*_p_ should be carried out by using the following expression: *n*_p_ = 5 × 1.6 *N*_p_ = 8.0∙*N*_p_.

**Figure 14:**
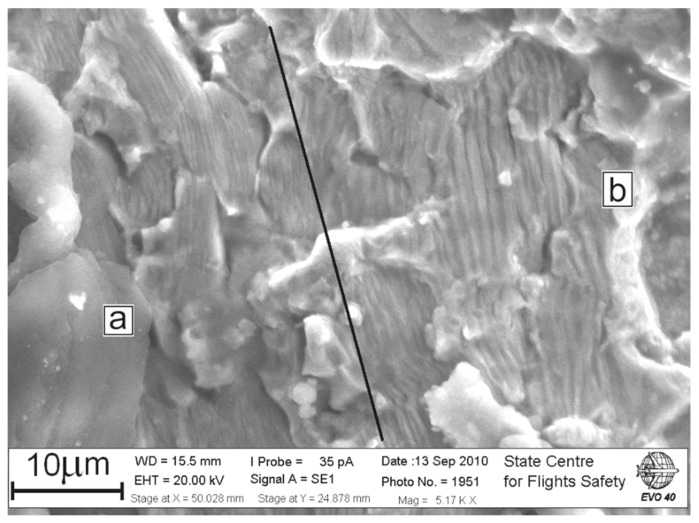
Fracture surface with two blocks “a” and “b” of fatigue striations with similar average spacing in each block, but differing by more than three times for the blocks “a” and “b”.

The validity of considering several loading cycles of the disk, causing the crack propagation during flight, is confirmed by the results of fractographic studies, presented, for example, in Figure 14. Within the fracture surface region length of about 40 µm, there are two blocks of fatigue striations “a” and “b” with an average spacing of 0.36 and 1.2 µm, respectively. In some fracture zones, pronounced blocks of fatigue striations formed without changing their spacing within sequences of striations belonging to one block (8–14 striations) were observed. These sequences can be a result of material fracture during the implementation of transient loading modes, as follows from the scheme of engine operating conditions for a typical flight (see Figure 13).

**Figure 15:**
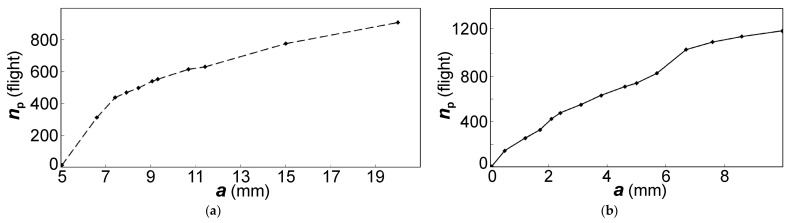
Dependences of the fatigue crack propagation duration in terms of flights *n*_p_ on the crack length *a* in the second-stage disk of the intermediate-pressure compressor (IPC) for directions (**a**) 1 and (**b**) 2 (shown in Figure 10).

The performed recalculation and the described regularities of disk fracture allowed obtaining the following results (Figure 15): (1) the crack growth duration along the direction 2 was about 1200 flight cycles; (2) the fracture durations in the directions 2 and 1 correspond to each other; and (3) within the range of first 5 mm along the surface of the slot for the blade, *n*_p_ of about 340 flight cycles should be considered (i.e., *n*_p_ = (9600–6900)/8). The dependencies shown in Figure 15 allow determining the frequency of disk inspections, starting with the crack length determined by the resolution of the inspection tool and the accessibility of the zone where the crack can be detected.

Thus, the obtained results of estimating the crack growth duration in terms of flight cycles using a ratio of eight unit cycles per one flight are quite reliable.

### 4.3. Summarizing the Results of the Disk Study

The results obtained in the study of the disk material quality and the established regularities of a fatigue crack growth indicate that the initiation of fatigue cracks occurred in a material that did not possess a deviation in the manufacturing quality of the second-stage disk of the IPC. Nevertheless, the concentration of the crack initiation zone localized within the radius transition, the orientation of the initial fracture zone boundary along the surface of the radius transition, and the LCF corresponding fracture surface peculiarities are evidence of the high-stress intensity of the disk in the fracture initiation zone. To identify the nature of such stress intensity, the history of engine operation and overhaul was analyzed.

Analysis of data on the operation and overhaul of the engine, including the second-stage disk of IPC under investigation, showed the following. At the initial stage of engine operation, the first-stage disk was replaced, which could affect the stress state of the second-stage disk, since they are welded into one drum of the compressor. The replacement could not fully reproduce the stress-state conditions that were realized during the initial manufacturing of the drum with the second-stage disk of the IPC. Later, during the second overhaul, a cascade of fluorescence was observed in the second-stage disk after an operating time of 2858 h and 5 min (or 539 FC). Fluorescence was detected in the radius transition to the bottom of the dovetail slot along a length of 5 mm, as well as from the side of the entrance and an acute angle in many locations of the disk. This implies the certain relaxation processes of stress state in the second-stage disk, which was created in the disk by welding to the cascade of the first-stage disk of the IPC in the first overhaul.

During the overhaul, a thin layer of disk material was removed to eliminate the fluorescence (assuming material inhomogeneities). On the one hand, this led to the elimination of possible material inhomogeneities, but, on the other hand, it did not remove the total changed stress state of the disk caused by welding of the first-stage disk of the IPC.

The analysis of regularities of fatigue crack initiation and growth in the second-stage disk of IPC showed that in the region of crack initiation, the fatigue striation spacing was reached 0.6 µm (see Figure 12). This indicates a high-stress intensity of the disk precisely in the initial fracture zone, where the multi-focal origin of the fracture occurred. The subsequent fracture development occurred under varying disk stress state conditions that follow from the analysis of the regularity of the fatigue crack propagation. Despite the significant intensity of the initial crack growth stage, further fracture development took place with insignificant acceleration, resulting in a long crack growth duration. This fact implies that the disk stress intensity decreased in the direction of crack growth. The geometry of the initial fracture zone confirms the existence of the extremely high-stress state of the disk material in the zone that is oriented along the radius transition to the slot bottom within 5 mm. Then, the crack kinked, and its orientation became almost perpendicular to the radial force (see Figure 10), i.e., to the total stress state realized in the disk during in-service loading. Moreover, the analysis of the boundary between the fracture zone with the region of radius transition to the slot bottom showed that it is oriented along the machining scores of the slot surface. At a high-stress intensity of the material, the radius transition plays a key role in creating a stress concentration, rather than the scores along this surface that are insignificant in depth (<5 μm). Their main influence was revealed in the formation of the crack initiation zone along the scores on the radius transition surface; i.e., there was a slight increase in the stress concentration along the specified surface.

Thus, it can be concluded that the failure of the second-stage disk of the IPC of the gas turbine engine has resulted from its high-stress intensity in the region of the radius transition to the slot bottom. Most probably, the indicated stress state resulted from welding of the first-stage disk of IPC after an operating time of 1985 h and 20 min (342 FC). At the operating time of 2858 h and 5 min (539 FC), the initial origins of the multiple non-uniform states in the second-stage disk detected during non-destructive testing were removed in the overhaul. This led to a decrease in the stress intensity in the region of the radius transition zone to the bottom of the slot but did not eliminate it.

The recommended inspection intervals for a disk with a developing fatigue crack were evaluated in accordance with the existing standard [1]. It follows from the standard that when introducing periodic monitoring, it is necessary to calculate the duration of the operation period between two adjacent inspections *n*_insp_ based on the fractographic assessment of the crack growth duration *n*_p_ in terms of flight cycles by using the expression:

(1) $$n_{\mathrm{insp}}=\frac{2}{3}\times \frac{n_{p}}{K_{N}},$$

where the value of factor *K*_N_ is varied from 2–4. Only in special cases, with a statistically reliable substantiation of the crack propagation duration *n*_p_ based on several cases of disk failure, the value of the factor can be reduced up to 2. In the considered situation with a single case of disk failure, a statistically reliable estimation of the crack growth duration is impossible. Therefore, the inspection intervals for the second-stage disk of the IPC should be considered with the application of *K*_N_ = 4 as a reduction factor, and the value of the inspection interval *n*_insp_ = (2/3) × (900/4) = 150 FC with an inspection method resolution of about 2 mm (see Figure 15). For an average flight time of 5 h and a flight frequency of once every three days, the duration of engine operation between inspections is of 750 h or 450 days, which guarantees a stable operation for a long period. With an increase in test-sensitivity, the inspection interval can be extended.

Thus, based on fractographic analysis of the regularities of fatigue crack growth, taking into account the effect of fatigue crack meso-tunneling, the inspection intervals for disks in service on the entire fleet of An-124 aircraft are established and substantiated, with ensuring the required level of flight safety, which are at least 150 flights. As information concerning the in-service inspections of disks, the crack detections and the estimation of the crack growth duration will be accumulated, the in-service duration between two adjacent inspections of the disk can be increased.

## 5. Conclusions

1. A VT3-1 titanium alloy with an inhomogeneous filament type texture obtained as a result of forging was studied in the LCF regime on specimens manufactured from compressor disks under loading conditions with triangular and trapezoidal cycle waveform. The considered structure of the material is formed at forging the compressor disks as a result of the wave process of plastic deformation, leading to the creation of alternating layers of metal with low and sufficiently high plasticity.
2. It is established that depending on the orientation of the fatigue crack propagation, a different fracture surface relief can be realized under loading conditions: in the case of crack growth perpendicular to the filaments, alternating regions of fatigue striations and quasi-brittle waved pattern are formed; in the case of crack growth along the material filaments, the process of crack meso-tunneling occurs.
3. Meso-tunneling of a crack is expressed in the initial fracture of the material along the brittle filaments with the formation of a facetted pattern relief on the fracture surface evidencing the two-phase structure of the titanium alloy, and then the bridges between the adjacent meso-tunnels are fractured with the formation of a block of fatigue striations.
4. The part of the fatigue crack growth duration *N*_p_/*N*_f_ is characterized by different dependences on the durability *N*_f_ in the case of crack propagation along or across the filaments of a forged titanium alloy.
5. Based on the analysis of the fracture process of the titanium second-stage disk of the intermediate-pressure compressor of the engine, it is shown how the effect of a crack meso-tunneling determines the regularities of fatigue crack propagation under implemented in-service conditions.
6. The duration of the fatigue crack propagation was estimated by measuring the fatigue striation spacing, and the consideration of the requirement to introduce a reduction factor when recalculating the number of unit loading cycles of the disk into the number of flight loading cycles was discussed, and a recommendation on a duration of the inspection intervals for the disk in service was given as well.
7. The reason for the initiation of a fatigue crack in the compressor disk is considered on the basis of data on its operation and taking into account the welded structure of the rotor of the IPC.

## Figures and Tables

**Table 1:** The results of the atomic emission spectroscopic analysis of the disk specimen in comparison with the main alloying elements of the grade composition in wt %.

Element/ Specimen	Ti	Al	Mo	Cr	Si	Fe
Disk	balance	6.0	2.5	2.0	0.4	0.5
VT3-1 grade	balance	5.5–7.0	2.0–3.0	0.8–2.3	0.15–0.40	0.2–0.7

## Data Availability

The data presented in this study are available on request from the corresponding author.

## References

[1] Shanyavskiy A. (2003). Tolerance Fatigue Failures of Aircraft Components. Synergetics in Engineering Applications.

[2] Williams J.C., Starke E.A. (2003). Progress in Structural Materials for Aerospace Systems. Acta Mater.

[3] McEvily A.J. (2004). Failures in inspection procedures: case studies. Eng. Fail. Anal..

[4] Shanyavskiy A.A., Stepanov N.V. (1995). Fractographic Analysis of Fatigue Crack Growth in Engine Compressor Disks of Ti-6Al-3Mo-2Cr Titanium Alloy. Fatigue Fract. Engng Mater. Struct..

[5] Shanyavskiy A.A., Losev A.I. (1999). Fatigue Crack Growth in Aroengine Compressor Disks Made from Titanium Alloy. Fatigue Fract. Engng. Mater. Struct..

[6] Shanyavskiy A.A., Losev A.I., Banov M.D. (1998). Development of Fatigue Cracking in Aircraft Engine Compressor Disks of Titanium Alloy Ti-6Al-3Mo-2Cr. Fatigue Fract. Eng. Mater. Struct..

[7] Wanhill R.J.H., Oldersma A., Beynon J.H., Brown M.W., Lindley T.C., Smith R.A., Tomkins B. (1999). Fatigue and Fracture in an Aircraft Engine Pylon. Engineering Against Fatigue, Sheffield, UK, 17–21 March 1997.

[8] Howard I.C., Hudson C., Rich T. (1986). Fracture of an Aircraft Horizontal Stabilizer. Case Histories Involving Fatigue and Fracture Mechanics, Charleston, SC, USA, 21–22 March 1985.

[9] Pilchak A.L., Williams J.C. (2010). Observations of Facet Formation in Near-α Titanium and Comments on the Role of Hydrogen. Metall. Mater. Trans. A.

[10] Kocańda D., Kocańda S., Tomaszek H., Pluvinage G., Gjonaj M. (2001). Probabilistic Description of Fatigue Crack Growth in a Titanium Alloy Notched Member. Notch Effects in Fatigue and Fracture. NATO Science Series II: Mathematics, Physics and Chemistry.

[11] Pilchak A.L. (2013). Fatigue Crack Growth Rates in Alpha Titanium: Faceted vs. Striation Growth. Scr. Mater..

[12] Shanyavskiy A.A., Losev A.I., Ellyin F., Provan J.W. (1999). Synergistic Problem of Introduction of Tolerance Damage Service of Titanium Disks of Aircraft Engines. Progress in Mechanical Behaviour of Materials. Vol. III: Advanced Materials and Modelling of Mechanical Behaviour, Proceedings of the Eighth International Conference on the Mechanical Behaviour of Materials (ICM8), Victoria, BC, Canada, 16–21 May 1999.

[13] Woodfield A.P., Gorman M.D., Corderman R.R., Sutliff J.A., Blenkinsop P.A., Evans W.J., Flower H.M. (1996). Effect of Microstructure on Dwell Fatigue Behavior of Ti-6242. Proceedings of the Eighth World Conference on Titanium (Titanium ’95: Science and Technology).

[14] Shanyavskiy A.A. (2005). The Effects of Loading Waveform and Microstructure on the Fatigue Response of Ti–6Al–2Sn–4Zn–2Mo Alloy. Fatigue Fract. Eng. Mater. Struct..

[15] (2019). ASTM E606 / E606M-19e1, Standard Test Method for Strain-Controlled Fatigue Testing.

[16] Shanyavskiy A.A., Burchenkova L.M. (2013). Mechanism for Fatigue Striations as Formed under Variable Negative R-ratio in Al-based Structural Alloys. Int. J. Fatigue.

[17] Jha S.K., Larsen J.M., Allison J.E., Jones J.W., Larsen J.M., Ritchie R.O. (2007). Random Heterogeneity Scales and Probabilistic Description of the Long-Lifetime Regime of Fatigue. Proceedings of the Fourth International Conference on Very High Cycle Fatigue (VHCF-4), University of Michigan.

[18] Panin V.E., Egorushkin V.E., Panin A.V. (2010). The Plastic Shear Channeling Effect and the Nonlinear Waves of Localized Plastic Deformation and Fracture. Phys. Mesomech..

[19] De Los Rios E., Kandi A., Miller K.J., Brown M.W., Miller K., Brown M. (1985). A Metallographic Study of Multiaxial Creep-fatigue Behavior in 316 Stainless Steel. Multiaxial Fatigue. ASTM STP 853.

[20] Miller K. (1986). Creep and Fracture.

[21] Couper M.J., Neeson A.E., Griffiths J.R. (1990). Casting Defects and the Fatigue Behaviour of an Aluminium Casting Alloy. Fatigue Fract. Eng. Mater. Struct..

[22] Takahashi I., Yoshii T., Iidaka H., Fujii E., Matsuoka K. (1993). Fatigue Strength of Non-load-carrying Fillet Welded Joints: Effects of Weld Residual Stresses and Stress Concentration. Fatigue Fract. Eng. Mater. Struct..

